# Repeated Exposure to Dissection Does Not Influence Students' Attitudes towards Human Body Donation for Anatomy Teaching

**DOI:** 10.1155/2016/9251049

**Published:** 2016-04-13

**Authors:** Philip Maseghe Mwachaka, Pamela Mandela, Hassan Saidi

**Affiliations:** Department of Human Anatomy, College of Health Sciences, School of Medicine, University of Nairobi, P.O. Box 30197, Nairobi 00100, Kenya

## Abstract

The use of unclaimed bodies for anatomical dissection has been the main method of instruction at our institution. There is however a shortage of cadavers for dissection given the increase in the number of medical schools as well as in the number of students enrolling in these schools. This shortage could be mitigated by having voluntary human body donation programs. This study aimed at assessing the attitudes of medical students and surgical residents towards body donation for anatomy learning. We conducted an online survey involving 72 first-year medical students and 41 surgical residents at University of Nairobi who had completed one year of anatomy dissection. For the medical students, this was their first dissection experience while it was the second exposure for the surgery trainees. Most of the surgical trainees (70.7%) and medical students (68.1%) were opposed to self-body donation. This was mainly due to cultural (37%) and religious (20%) barriers. Surprisingly, of those not willing to donate themselves, 67.9% (82.8% surgical trainees, 59.2% medical students) would recommend the practice to other people. Exposure to repeated dissection does not change the perceptions towards body donation. It is noteworthy that culture and religion rank high as clear barriers amongst this “highly informed” group of potential donors.

## 1. Introduction

Human cadavers are important in the initial and continuing training of medical doctors and advancement of medical research [1–3]. Cadaveric dissection has been the main mode of learning anatomy for many medical schools [4, 5]. Whereas donated cadavers make up 80–100% of the total cadavers in European and North American medical schools, up to 90% of African medical schools still rely on unclaimed bodies [6–9]. In Kenya, all medical schools use unclaimed bodies obtained from public health facilities around the country [10]. At the University of Nairobi, the oldest medical school in Kenya, all the cadavers used for dissection are unclaimed bodies [10, 11]. This school runs a 6-year undergraduate bachelor of medicine and surgery course and a postgraduate surgical residency course. Students in these two groups spend at least 250 hours of their first year of study dissecting the human body.

With the increasing demand for more healthcare workers in the country, many medical schools have been set up. The number of medical schools in Kenya has grown from one in 1967 to nine in 2015, with each school having massive expansion in student enrollment. All these schools compete for the same pool of unclaimed bodies resulting in shortage of cadavers for dissection. For instance, despite increased medical student enrollment at the University of Nairobi to over 400 students per year, the supply of cadavers has stagnated at 50 per year. This cadaver shortage has led to increase in the cadaver to student ratio from 1 : 6 to 1 : 9.

Scarcity of unclaimed bodies for dissection necessitates development of human body bequest programs [9, 12, 13] especially in African countries which have solely been relying on unclaimed bodies for anatomy teaching [7, 10]. Although the University of Nairobi, Kenya, has a human body donation program, it hardly receives any donated bodies. For instance, only two bodies have been donated in the last year. This study therefore aimed at assessing the attitudes of the end-users (the students) towards body donation and whether a repeat exposure to cadaveric dissection would positively influence the student perceptions towards body donation.

## 2. Methods

### 2.1. Participants and Setting

This study enrolled first-year medical (undergraduate) students and surgical (postgraduate) residents at the University of Nairobi (UoN) in Kenya, after they had completed one year of cadaveric dissection. For the medical students, this was their first dissection experience while it was the second exposure for the surgery trainees, having dissected when they were undergraduate medical students. After ethical approval by the School of Medicine, University of Nairobi, an anonymous online based questionnaire was sent to 150 undergraduate and 55 postgraduate students. All participants were informed of the aims of the study, and their involvement was voluntary. This survey was conducted between 11 April, 2015, and 10 May, 2015. Seventy-two undergraduate (response rate 48%) and 41 postgraduate (response rate 74.5%) students completed the survey.

### 2.2. Questionnaire

Variables collected in the self-administered questionnaire included the course, gender, whether they would donate their bodies for anatomy teaching (yes/no answer), reasons for/against body donation (open-ended answer), whether they have heard of any local body donation program (yes/no answer), whether they would recommend body donation to other people (yes/no answer), and the reasons for recommending so (open-ended answer).

### 2.3. Statistical Analysis

Statistical analysis was done using the Statistical Package for the Social Sciences (SPSS) for Windows version 21.0 (SPSS Inc., Chicago, IL). The Chi-square test was used to compare the responses between the undergraduate (medical) and postgraduate (surgical) students. A *p* value <0.05 was considered significant.

## 3. Results

### 3.1. Awareness of a Local Body Donation Program

Of the 113 students surveyed, 24.8% had heard of the body donation program at the UoN. More of the postgraduate students (43.9%) compared to medical students (13.9%) were aware of the local body bequest program (Table 1). The Chi-square test posted a Pearson *χ* ^2^ = 12.626 and *p* < 0.001, indicating a highly statistically significant difference in the proportionate distribution of those who were aware of a local body donation program.

### 3.2. Willingness to Donate Their Bodies

**Figure 1:**
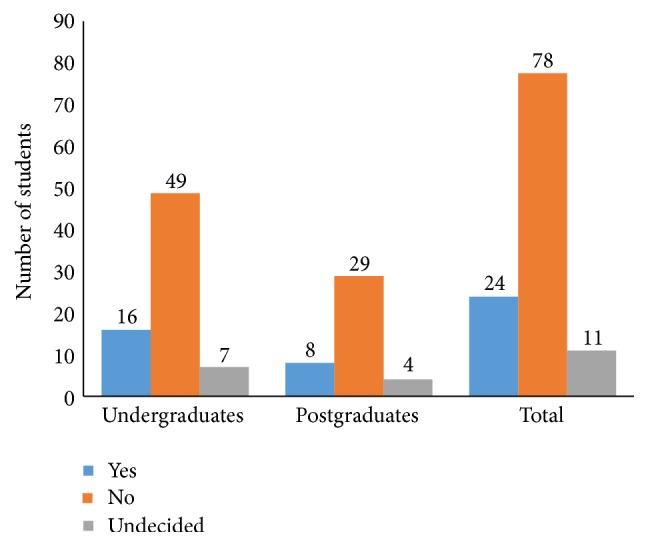
Proportions of students willing to donate their bodies for anatomy teaching.

Only 24 (21.2%) of the respondents were willing to donate their own bodies (Figure 1). Only 16 (22.2%) of the undergraduates and 8 (19.5%) of the postgraduates were willing to donate their own bodies (*p* = 0.943). It is noteworthy that 11 (9.7%) students were undecided on whether they would donate or not.

### 3.3. Reasons for Being Not Willing to Donate Their Bodies

Of those who were not willing to donate, 70 respondents gave their reasons which were mainly cultural (37.1%) and religious (20.0%) barriers (Table 2). Apart from cultural and religious reasons, 16 (22.8%) of the undergraduates felt that the amount of mutilation done at cadaveric dissection was too much. They decried the handling of cadavers especially by fellow students, terming it as undignified, uncomfortable, brutal, and sad. Three students felt the human body is sacred and should be treated as such. Two students thought that their relatives would not find closure if they donated their bodies. The postgraduates had similar thoughts. Five (17.2%) of these students decried the level of mutilation at the dissection laboratory and found the idea of students practicing on their bodies to be rather repugnant. Two of the students would consider body donation for professional development of surgical specialization skills training, arguing that this mature set of students would better appreciate the body donation than do undergraduates. Two postgraduates preferred organ donation to whole body donation as they felt that it saves lives.

### 3.4. Recommendation of Body Donation for Anatomy Teaching to Other People

**Figure 2:**
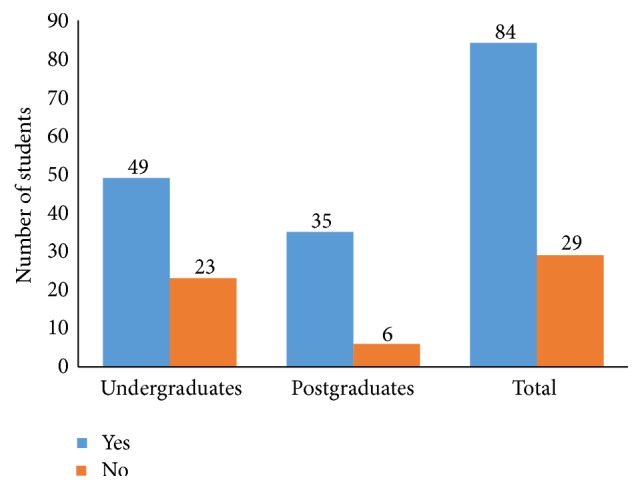
Recommendation of body donation to other people.

There were 84 (74.3%) of students who were willing to recommend body donation for anatomy teaching to other people (Figure 2). Significantly more postgraduate, 35 (85.4%), than undergraduate, 49 (68.1%), students would recommend body donation for anatomy teaching to other people (*p* = 0.043). Surprisingly, 53 (67.9%) respondents who were not willing to donate their own bodies would recommend body donation for teaching anatomy to other people (Table 3). Notably, more postgraduate students (82.8%), who were not willing to donate their bodies, will recommend the practice to others in comparison with the undergraduate students (59.2%) with a similar view (*p* = 0.031; Table 3).

### 3.5. Reasons for Recommending Body Donation to Other People

All the respondents who will recommend body donation to others noted that there was a need for more cadavers for teaching and research and this was the motivation for their recommendation. They also acknowledged cadaveric dissection as the best way to learn anatomy for future doctors. They however stated that potential donors should be provided with information on how the bodies will be handled before, during, and after dissection. In addition, the postgraduates recognized the need for body donation particularly for surgical skills training. They were also aware of the looming shortage of unclaimed bodies for cadaveric dissection.

## 4. Discussion

This study was seeking to determine the perception of undergraduate and postgraduate students towards body donation for learning anatomy. Body donation is an altruistic act which involves giving one's body after death for medical education and research [7]. The small proportion of students who are aware of a local body donation is an indicator that there is a lack of general information about the program. This could be due to lack of active campaigns about the importance of body donation for anatomy teaching. It is also possible that body donation as a source of cadavers for dissection may not have been emphasized to the students during induction to dissection classes, because all the cadavers used are from unclaimed bodies. This lack of knowledge of body donation programs is a barrier to becoming active donors [14–16]. Therefore, there is a need to educate both the students and the general public on the need and availability of body bequest programs [12, 17–20]. The students need to be involved right from the time they start their studies [9].

Unwillingness to enroll in body bequest program by medical students and professionals seen in our study has also been reported in other studies. For instance, only 13.5% of first-year medical students in France [21] and 6% and 2% of medical students and doctors, respectively, in India were willing to donate their bodies for dissection [22]. A study on first-year Irish Medical students reported decreased support for body donation by the students from 31.5% before dissection to 19.6% after dissecting for 9 weeks [23]. A study in India reported that only 22% of physicians were willing to donate their bodies for medical education, but 68% expected the public to donate [17]. Surprisingly, even anatomists themselves are not willing to donate their bodies [17, 24]. In these two studies, only 15.7% of Turkish [17] and 25% of Dutch [24] anatomists were willing to donate their bodies.

The reasons against self-body donation given in the current study, namely, religious beliefs and customs as well as poor handling of the bodies during dissection, have been reported in other studies [25, 26]. Studies in India have also reported firm religious beliefs and customs and the fear that the donated body will not be treated with respect and dignity as hindrances towards successful body donation programs [26]. Australian chiropractic students reported that atheistic and agnostic students were more willing to donate than religious students [25]. The same study also reported that willingness to donate one's own or a family member's body decreased as year of study increases, suggesting a possible negative impact of exposure to cadavers in the anatomy laboratory [25]. Other studies on the characteristics of body donors have also revealed that most people who registered as donors have no religious affiliations [13, 27, 28].

For a body bequest program to be successful, issues raised by the students need to be addressed. Clear guidelines need to be laid down on the handling of the cadavers from the time they are received, during dissections, and in final disposal of the remnants of dissections [29, 30]. Cadavers should be handled with respect and honor throughout the dissection period. Respectable gestures such as referring to the cadaver as the “silent teacher” rather than just an anatomy specimen have been shown to have a positive influence on the attitudes of medical students towards the cadaver [7, 31]. We should also borrow from other body bequest programs that have a dedication service before start of dissection and a thanksgiving or memorial service at the end of the dissection period [9, 29, 32, 33]. During the dedication services, the cadavers are dedicated to the training of the students, and the students are taught to value and respect the cadavers [9]. At the end of each dissection year, the general public, students, and staff come together to bid farewell to the donors in a decent burial ceremony [9, 29, 32, 33]. This helps reassure apprehensive prospective donors that their remains will be treated with dignity.

## 5. Conclusion

This study has demonstrated that whereas students expect to learn anatomy by dissecting, as a potential donor population, they are reluctant to become donors themselves. Exposure to repeated dissection does not have a positive influence on the perceptions towards body donation. Most are hindered by cultural and religious reasons from participating in the body donation program. Lack of awareness of local bequest program may be a major hindrance to body donation.

## Supplementary Material

Data collection tool (questionnaire) used to collect undergraduate medical and post graduate surgical students' views on human body donation for anatomy teaching.

## Figures and Tables

**Table 1:** Proportions of students aware of a body donation program at UoN.

Have you heard of any local body donation program?	Undergraduate	Postgraduate	Total
Yes	10 (13.9%)	18 (43.9%)	28 (24.8%)
No	62 (86.1%)	23 (56.1%)	85 (75.2%)
Total	72 (100.0%)	41 (100.0%)	113 (100.0%)

**Table 2:** Reasons for being not willing to donate their bodies.

Reasons	Undergraduate	Postgraduate	Total
Cultural	13 (31.7%)	13 (44.8%)	26 (37.1%)
Religious	7 (17.1%)	7 (24.1%)	14 (20.0%)
Others	21 (51.2%)	9 (31.0%)	30 (42.9%)
Total	41 (100.0%)	29 (100.0%)	70 (100.0%)

**Table 3:** Proportions of students not willing to donate their bodies but who would recommend body donation for anatomy teaching to other people.

Recommend body donation to other people?	Undergraduate	Postgraduate	Total
Yes	29 (59.2%)	24 (82.8%)	53 (67.9%)
No	20 (40.8%)	5 (17.2%)	25 (32.1%)
Total	49 (100.0%)	29 (100.0%)	78 (100.0%)
