# Supplementary material for: Repeated Exposure to Dissection Does Not Influence Students' Attitudes towards Human Body Donation for Anatomy Teaching
Source: Anat Res Int. 2016 Apr 13;2016:9251049. doi: 10.1155/2016/9251049 (PMC4846756; doi:10.1155/2016/9251049)
Supplement: Supplementary file 1 — Data collection tool (questionnaire) used to collect undergraduate medical and post graduate surgical students' views on human body donation for anatomy teaching. [file 9251049.f1.docx]

**MEDICAL STUDENTS PERCEPTION TOWARDS HUMAN BODY DONATION FOR ANATOMY TEACHING**

Dear student. This questionnaire is aimed at collecting your views on human body donation for anatomy teaching. Please answer all questions to the best of your ability. There are no ‘correct’ or ‘incorrect’ answers. All the data collected will be treated with utmost confidentiality and will in no way be used against you. Thanks for your cooperation and patience.

**Gender:** Male 🞏 Female🞏

**Course:** MBChB 🞏 POST GRADUATE (MMed) 🞏

1. **Have you heard of any local body donation program for anatomy teaching?**  Yes 🞏 No 🞏
2. **Would you be willing to donate your body for anatomy teaching?**

Yes 🞏 No 🞏 Undecided 🞏

**State the reasons for your response:**

**______________________________________________________________________________________________________________________________________________________________________________________________________________________________________________________**

**______________________________________________________________________________________________________________________________________________________________________________________________________________________________________________________**

**______________________________________________________________________________________________________________________________________________________________________________________________________________________________________________________**

**______________________________________________________________________________________________________________________________________________________________________________________________________________________________________________________**

1. **Would you recommend body donation for anatomy teaching to other people?**

Yes 🞏 No 🞏 Undecided 🞏

**State the reasons for your response:**

**______________________________________________________________________________________________________________________________________________________________________________________________________________________________________________________**

**______________________________________________________________________________________________________________________________________________________________________________________________________________________________________________________**

**______________________________________________________________________________________________________________________________________________________________________________________________________________________________________________________**
